# Metabolic Profiling of Primary and Secondary Metabolites in Kohlrabi (*Brassica oleracea* var. *gongylodes*) Sprouts Exposed to Different Light-Emitting Diodes

**DOI:** 10.3390/plants12061296

**Published:** 2023-03-13

**Authors:** Ramaraj Sathasivam, Sang Un Park, Jae Kwang Kim, Young Jin Park, Min Cheol Kim, Bao Van Nguyen, Sook Young Lee

**Affiliations:** 1 Department of Crop Science, Chungnam National University, 99 Daehak-ro, Yuseong-gu, Daejeon 34134, Republic of Korea; 2 Department of Smart Agriculture Systems, Chungnam National University, 99 Daehak-ro, Yuseong-gu, Daejeon 34134, Republic of Korea; 3 Division of Life Sciences and Convergence Research Center for Insect Vectors, College of Life Sciences and Bioengineering, Incheon National University, Yeonsu-gu, Incheon 22012, Republic of Korea; 4 Marine Bio Research Center, Chosun University, 61-220 Myeongsasimni, Sinji-myeon, Wando-gun 59146, Republic of Korea

**Keywords:** LED lights, kohlrabi sprouts, *Brassica oleracea*, carotenoid, glucosinolates, phenylpropanoid, hydrophilic compounds

## Abstract

Light-emitting diode (LED) technology is one of the most important light sources in the plant industry for enhancing growth and specific metabolites in plants. In this study, we analyzed the growth, primary and secondary metabolites of 10 days old kohlrabi (*Brassica oleracea* var. *gongylodes*) sprouts exposed to different LED light conditions. The results showed that the highest fresh weight was achieved under red LED light, whereas the highest shoot and root lengths were recorded below the blue LED light. Furthermore, high-performance liquid chromatography (HPLC) analysis revealed the presence of 13 phenylpropanoid compounds, 8 glucosinolates (GSLs), and 5 different carotenoids. The phenylpropanoid and GSL contents were highest under blue LED light. In contrast, the carotenoid content was found to be maximum beneath white LED light. Principal component analysis (PCA) and partial least-squares discriminant analysis (PLS-DA) of the 71 identified metabolites using HPLC and gas chromatography–time-of-flight mass spectrometry (GC-TOF-MS) showed a clear separation, indicating that different LEDs exhibited variation in the accumulation of primary and secondary metabolites. A heat map and hierarchical clustering analysis revealed that blue LED light accumulated the highest amount of primary and secondary metabolites. Overall, our results demonstrate that exposure of kohlrabi sprouts to blue LED light is the most suitable condition for the highest growth and is effective in increasing the phenylpropanoid and GSL content, whereas white light might be used to enhance carotenoid compounds in kohlrabi sprouts.

## 1. Introduction

Kohlrabi (*Brassica oleracea* var. *gongylodes*) is a biennial plant that belongs to the Cruciferae family (*Brassicaceae*) [1]. It is a *Brassica oleracea* species, similar to cabbage, cauliflower, broccoli, and kale. Round and swollen stems of kohlrabi are mainly used by human beings for food [2,3]. Depending on the color, it is classified as purple or pale green kohlrabi; however, the color of the flesh is the same as that of white [4,5]. Only purple kohlrabi contains anthocyanins and has a higher phenylpropanoid content than green kohlrabi [6]. Various studies have shown cruciferous vegetables, including kohlrabi, are beneficial for human health, as they contain abundant ascorbic acid, carotenoids, and tocopherol [7,8].

Sprouts are produced by seed germination and are in a state after the cotyledon emerges and before the primary leaves emerge [9]. During germination, sprouts synthesize nutrients and physiologically active substances for growth [9]. They are prominent sources of vitamins, minerals, and proteins for human health and contain phenolic compounds, selenium-containing compounds, and GSLs [10,11]. Cruciferous sprouts are attracting attention as they contain more phenylpropanoids and GSLs than complete plants [12,13,14]. According to a previous study, the flesh of red kohlrabi contains eight types of GSLs, and among them, glucoerucin is the most abundant compound [6].

Phenylpropanoids are a diverse group of organic compounds formed from the combination of the amino acids phenylalanine and tyrosine and are synthesized in plants [15,16]. Several reports have revealed that phenylpropanoids have beneficial effects on human health and the pharmaceutical industry [17,18]. For instance, phenylpropanoids help prevent cancer, diabetes, and heart disease [19,20]. Glucosinolates (GSLs) are important secondary metabolites that contain nitrogen and sulfur in their structure and can be classified into aliphatic, aromatic, and indolyl GSL based on their chemical structure [21,22]. GSLs exhibit strong antioxidant and anticarcinogenic activities in humans [23,24]. Carotenoids are a group of natural pigments present in plants and animals that are responsible for orange, yellow, and red colors; improve photosynthesis in plants; and act as photo protectors by quenching reactive oxygen species [25,26,27,28]. Carotenoids have antioxidant and UV protective effects and aid in vitamin synthesis [27,29]. Therefore, they can improve human health and help prevent conditions such as blindness, skin aging heart disease, and cancer [30,31].

Light is the major constituent of plants and affects photosynthesis, physiology, and growth. It is also associated with secondary metabolite production [32]. Light-emitting diodes (LEDs) are light-emitting semiconductor devices that emit light only when they pass forward voltage. LEDs can produce various colors and wavelengths depending on the material. In addition, they have the advantages of a long lifetime, small size, and low emission temperature [33]. Many studies have been conducted on the relationship between LEDs and secondary metabolites in *Triticum aestivum* L. sprouts [32], *Panax ginseng* Meyer [34], *Agsatache rugosa* [35], *Brassica napus* L. [36], *Fagopyrum tataricum* [37,38], *Brassica rapa* ssp. *pekinensis* [39,40], *Vigna unguiculata* [41], and *Scutellaria baicalensis* [42]. However, the metabolic profiles of primary and secondary metabolites in *B. oleracea*, especially in *gongylodes* variety sprouts have not been investigated after exposure to various LEDs.

Thus, it is shown that various LEDs can have a significant effect on the primary and secondary metabolic content of many plant species. This study aims to investigate the effect of four different light wavelengths of white (449–551 nm), red (636 nm), blue (452 nm), and red + blue (457 and 636 nm). LEDs light on the growth and production of phenylpropanoid, carotenoid, and GSL in the 10 days old kohlrabi sprouts. This study may provide insight to researchers for enhancing the plant growth and accumulation of primary and secondary metabolites by selecting a suitable LED light in red kohlrabi sprouts. In addition, this present research will be helpful for the researchers to manipulate the primary and secondary metabolites concentration within the *B. oleracea* especially in *gongylodes* variety through changes in the LED light.

## 2. Results and Discussion

### 2.1. Effect of LED Light Treatment on the Growth of Kohlrabi Sprouts

**Figure 1:**
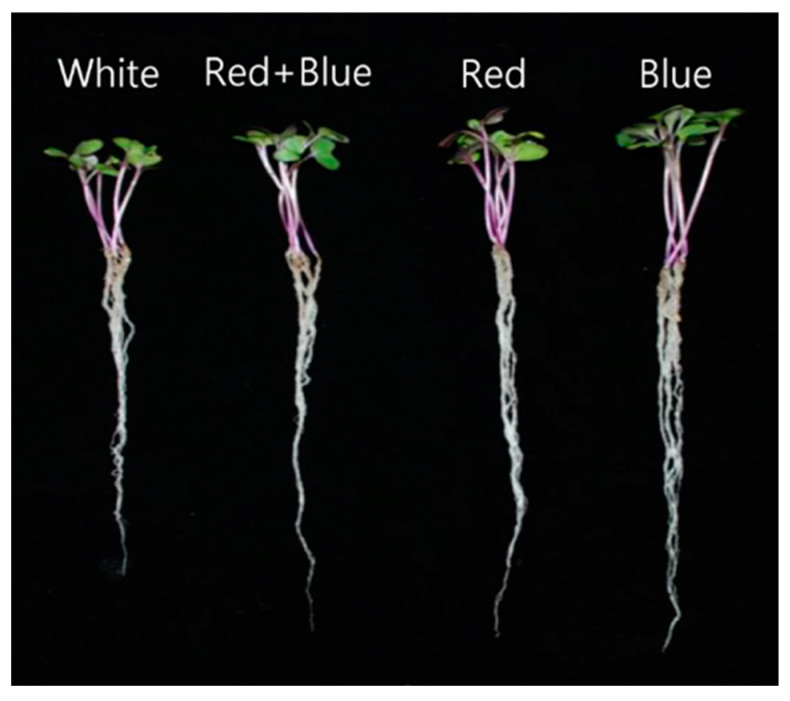
Development of kohlrabi sprout at 10 days of growth under white, blue, red, and blue + red LED lights.

**Figure 2:**
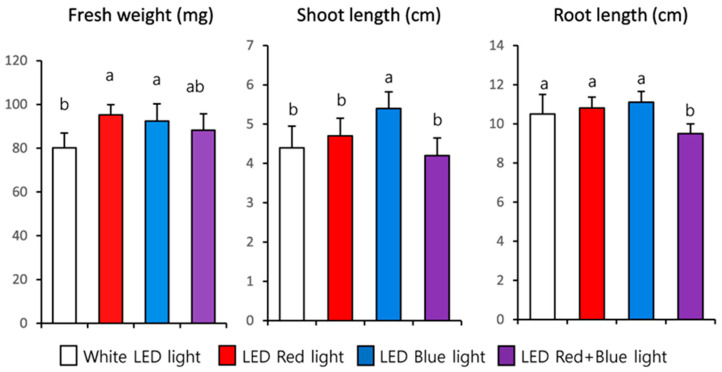
Effect of white, blue, red, and red + blue LED lights on the growth of 10 days for kohlrabi sprouts. Mean values marked with different alphabetical letters were significantly different (*p* < 0.05, ANOVA, DMRT).

LED light significantly influenced all growth parameters (Figure 1 and Figure 2). The red LED exhibited the highest fresh weight (95.20 mg), followed by the blue LED (92.40 mg) and red + blue LED (88.20 mg), whereas the lowest was achieved in the white LED treatment (80.20 mg). In contrast, the highest shoot length was recorded by the blue LED (5.40 cm), followed by the red LED (4.70 cm), white LED (4.40 cm), and red + blue LED (4.20 cm) light treatments. The trend of root length was similar to that of shoot length; the highest root length was recorded in the blue LED (11.10 cm) treatment, followed by the red LED (10.80 cm) and white LED (10.50 cm) treatments, whereas the lowest shoot length was observed in the red + blue LED (9.50 cm) light treatment.

It has already been proven that LEDs have significant advantages over artificial light sources such as fluorescent, high-pressure sodium lamps, and metalhalides for the plant in a controlled environment [43,44,45]. Light quality (e.g., reduced R/Fr ratio), decreased photosynthetically active radiation, and intensity directly influence the growth, morphogenesis, plant development, and production of secondary metabolites [46,47,48,49,50]. Red LED light is suitable for the growth of canola sprouts [36]. In addition, when *Brassica juncea* sprout was grown under the red light for three weeks, the dry weight (DW) was increased compared to the white light exposed sprout [51]. Thew et al. [37] reported that the fresh weight of *F. tataricum* sprouts was significantly increased under red LED light when compared with blue and white LED light irradiation. Our findings are consistent with previous studies reporting that the red LED showed the highest fresh weight. The results showed that the highest shoot lengths were recorded in the blue LED treatment compared to those in the other LED light exposure treatments. This result is consistent with that of a previous study that showed that blue LED light prevents unfavorable morphological changes, which stimulate hypocotyl/stem elongation in mustard plants [52]. Li et al. [53] reported that blue LEDs enhanced the vegetative growth of non-heading Chinese cabbage [54]. Similarly, exposing cucumber seedlings to monochromatic blue light enhanced hypocotyl growth, indicating that there is a species-specific difference [55]. In another study where *Polygonum tinctorium* was exposed to various LED light conditions, monochromatic blue LED light at the 24-h photoperiod increased the length of plantlet main stems faster than monochromatic red LED light. This result showed that prolonged exposure of plantlets to blue LED light increased stem growth. Similarly, prolonged exposure of cos lettuce to blue LED light increased leaf elongation, probably due to the phytochrome reaction [56,57,58]. Photoreceptor cryptochromes have been reported to be responsible for plant height, and they exhibit maximal activity when activated with blue light [59,60]. Blue LEDs may stimulate cryptochrome photoreceptors, thus resulting in enhanced shoot length in the plantlets exposed to blue LEDs. These results show that red and blue LEDs are suitable for the fresh weight. Therefore, in *B. oleracea*, blue LEDs improve shoot length, whereas white, red, and blue LEDs improve root length.

### 2.2. Accumulation of Phenylpropanoid Content in Kohlrabi Sprouts Irradiated with Different LED Lights

**Figure 3:**
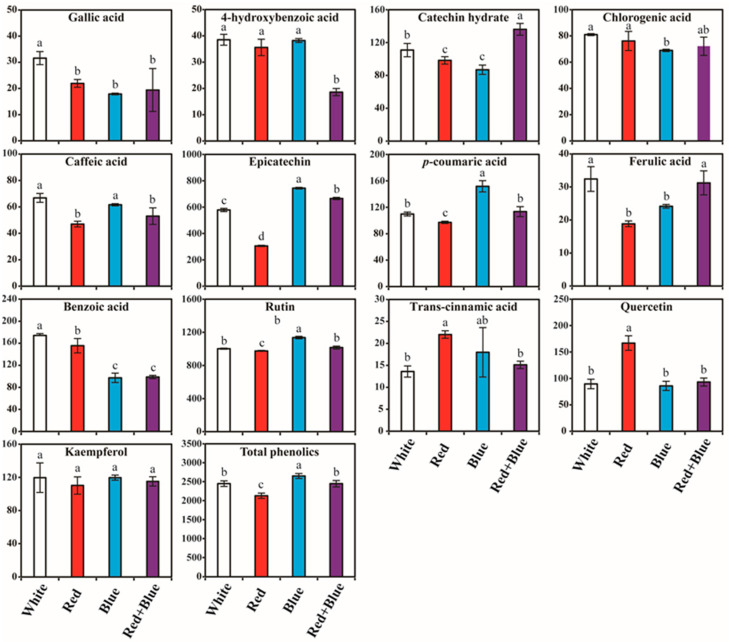
Phenylpropanoid content (µg/g DW) in kohlrabi sprouts irradiated with different LED lights. Samples were collected after 10 days of growth. Mean values marked with different alphabetical letters were significantly different (*p* < 0.05, ANOVA, DMRT).

The individual and total phenolic compound contents varied under different LED light conditions (Figure 3 and Table S1). The range of total phenylpropanoid content varied from 2129.7 to 2649.5 µg/g DW. The highest total phenylpropanoid content (µg/g DW) was achieved in blue LED light (2649.5), whereas the lowest was achieved in red LED light (2129.7) treatment. In addition to the total phenylpropanoid content, blue LED light treatment enhanced the accumulation of individual phenylpropanoid compounds. The trend of individual phenylpropanoid accumulation in the blue light exposed sprout ranged from highest to the lowest were as follows: rutin, epicatechin, *p*-coumaric acid, kaempferol, benzoic acid, catechin hydrate, quercetin, chlorogenic acid, caffeic acid, 4-hydroxybenzoic acid, ferulic acid, trans-cinnamic acid, and gallic acid, and their values were 1135.77, 743.60, 151.71, 119.62, 97.28, 86.87, 85.82, 68.95, 61.65, 38.21, 24.15, 17.97, and 17.88 µg/g DW, respectively. Sprouts exposed to white LED light showed the highest amounts of gallic acid, 4-hydroxybenzoic acid, chlorogenic acid, caffeic acid, ferulic acid, and benzoic acid. The total phenylpropanoid content was the lowest under the red LED light condition, where the highest amounts of trans-cinnamic acid and quercetin were detected. The level of catechin hydrate was the highest under red + blue LED light conditions. In all LED light exposures, the accumulation of rutin and epicatechin was significantly higher than that of the other compounds. The contents of rutin in the different LED light treatments ranged from 974.60 to 1135.77 µg/g DW (Figure 3). The highest amount of rutin accumulated in the blue LED light (1135.77 µg/g DW), and the lowest content was achieved in the red LED treatment. The second-most prevalent compound was epicatechin. The contents of epicatechin in the different LED light conditions ranged from 305.63 to 743.60 µg/g DW. The epicatechin content was higher in the blue LED light treatment, whereas the red + blue, white, and red LED light condition showed the lowest content as follows: 665.32, 578.45, and 305.63 μg/g DW, respectively. The third most prevalent compound is benzoic acid. The benzoic acid content in the LED light treatments ranged from 97.28 to 174.13 μg/g DW (Figure 3). The highest benzoic acid was achieved in white LED followed by red, red + blue, and blue LED light, and the levels were 174.13, 155.50, 98.87, and 97.28 μg/g DW, respectively. Among the individual phenylpropanoid compounds, trans-cinnamic acid had the lowest content was obtained in the white LED. The contents of trans-cinnamic acid in the LED light treatments ranged from 13.58 to 22.01 μg/g DW. The highest trans-cinnamic acid content was found in the red LED light treatment, followed by the blue, red + blue, and white LED treatments. The overall results showed that the exposure of red kohlrabi for 10 days to blue and white LED light accumulated the highest amount of total and individual phenylpropanoid compounds in red kohlrabi. 

In this study, the total and individual phenylpropanoid compounds were highest in the kohlrabi sprouts irradiated with blue LED, followed by white LED light. These results were consistent with previous findings that the exposure of Chinese cabbage seedlings, cowpea sprouts, and ginseng adventitious roots to blue LED light resulted in a marked increase in phenolic compounds [40,41,61]. In addition, irradiation of tartary buckwheat sprouts with blue LED light upregulated phenylpropanoid pathway genes and enhanced the phenolic compound production [37]. Moreover, *Aronia arbutifolia*, *Aronia melanocarpa*, and *Aronia prunifolia* shoot cultures [62] and *Peucedanum japonicum* Thunb. callus exposed to blue LED light increased the total phenolic content [63], whereas exposure of *Ocimum basilicum* callus led to the prominent aggregation of chlorogenic acid [64]. Several studies have reported that blue light increases the accumulation of secondary metabolites in Norway spruce [65], lettuce [66], and Chinese cabbage [40]. In another study, when comparing the white light, blue light improved the accumulation of quercetin and gallic acid content, whereas the level of *p*-coumaric acid and epicatechin was decreased in wheat sprouts [32]. Park et al. [35] reported the highest accumulation of rosmarinic acid and tilianin in *Agastache* rugosa plantlets exposed to white LED lights. Similarly, tartary buckwheat sprouts grown under fluorescent light showed an enhanced accumulation of rutin compared to the other phenolic compounds [67]. A previous study claimed that red light increased the ferulic acid and *p*-coumaric acid accumulation on days 8 and 12 of wheat sprout exposures [32]. These studies suggest that different LED lights positively impact the accumulation of pharmaceutically important secondary metabolites. These results might help researchers improve the level of specific pharmaceutical compounds in red kohlrabi sprouts by growing them under suitable LED light conditions. 

### 2.3. Accumulation of Glucosinolate in Kohlrabi Sprouts Irradiated with Different LED Lights

**Figure 4:**
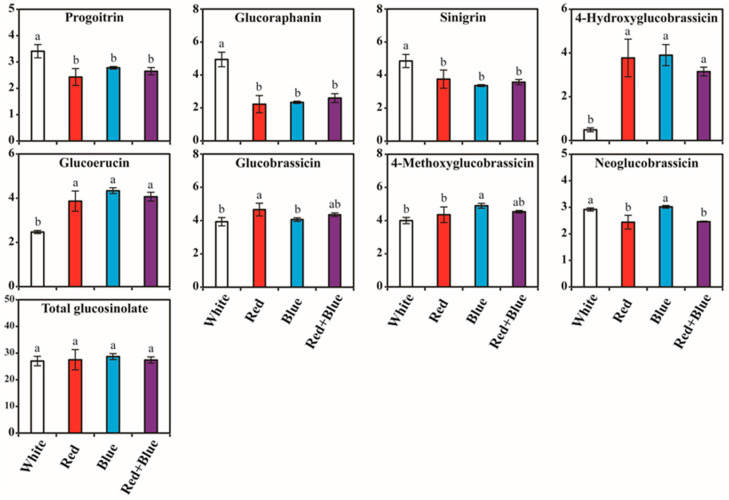
Glucosinolate contents (µg/g DW) in kohlrabi sprouts irradiated with different LED lights. Samples were collected after 10 days of growth. Mean values marked with different alphabetical letters were significantly different (*p* < 0.05, ANOVA, DMRT).

The individual GSLs compounds varied significantly under LED light conditions, whereas the total GSLs content did not differ significantly after LED light treatment (Figure 4 and Table S2). The range of total GSLs content varied from 27.01 to 28.69 µg/g DW. The highest total GSLs content (µg/g DW) was achieved with blue LED (28.69), followed by red (27.50), red + blue (27.38), and white (27.01). The individual GSL contents, such as 4-hydroxyglucobrassicin, glucoerucin, 4-methoxyglucobrassicin, and neoglucobrassicin, were also increased in kohlrabi sprout exposed to blue LED light. In blue LED light treatment, the level of 4-hydroxyglucobrassicin content was highest in the following orders: blue, red, red + blue, and white LED light, and the respective GSL values were 3.90, 3.77, 3.15, and 0.49 µg/g DW, respectively. In addition, blue LED light exposure enhanced the accumulation of the glucoerucin content, which was 1.76-, 1.12-, and 1.07-times higher than that in the sprouts exposed to white, red, and red + blue LED light, respectively. A higher 4-methoxyglucobrassicin content was noted in the blue LED light treatments, whereas the other LEDs exposures did not show much difference in their contents. A similar trend was observed in the neoglucobrassicin content of kohlrabi sprouts exposed to different LED lights. Although the total GSLs content was the lowest under white LED light conditions, individual GSLs, such as progoitrin, glucoraphanin, and sinigrin, were high. The contents of progoitrin in the treatments of different LED lights ranged from 2.43 to 3.41 μg/g DW (Figure 4). The highest level of progoitrin (μg/g DW) was attained under white light (3.41), followed by blue (2.78), red + blue (2.65) and red (2.43) LED light conditions. The glucoraphanin contents varied significantly between white LED and other LED conditions. The contents of glucoraphanin (4.94 μg/g DW) were 2.22-, 2.11-, and 1.9-times higher than the red, blue, and red + blue LED light-exposed conditions, respectively. Similarly, the sinigrin content was also higher in the white LED light exposure than in the other LED light exposures. The highest amount of glucobrassicin was observed under red LED irradiation. Under the red + blue LED light condition, none of the individual GSLs showed the highest content when compared to the other LED exposures. Red LED light was used to enhance the accumulation of glucobrassicin. Glucobrassicin in the treatments of LED light ranged from 3.93 to 4.66 μg/g DW. The glucobrassicin was the highest in the red LED light treatment, which was 1.19-, 1.15-, and 1.07-times higher than that of the sprouts exposed to white, blue, and red + blue LED light, respectively. 

It is well known that Brassica vegetables are rich in GSLs. In the present investigation, we detected and quantified eight individual GSL compounds, 4-hydroxyglucobrassicin, 4-methoxyglucobrassicin, glucobrassicin, glucoerucin, glucoraphanin, neoglucobrassicin, progoitrin, and sinigrin, which varied significantly under different LED light conditions. These results are consistent with those of previous studies that in rapeseed (*Brassica napus* L.); they identified six individual GSL compounds, such as 4-hydroxyglucobrassicin, glucobrassicin, glucobrassicanapin, gluconapin, gluconasturtiin, and progoitrin [68,69,70]. Similarly, the phytochemical analysis of *B. napus* and *Brassica oleracea* leaves identified 11 GSL compounds, including 4-hydroxyglucobrassicin, glucobrassicin, glucobrassicanapin, glucoiberin, gluconapin, gluconapoleiferin, gluconasturtiin, glucoraphanin, neoglucobrassicin, progoitrin, and sinigrin [71]. Park et al. [36] reported that *B. napus* sprouts exposed to different LED lights, such as blue, red, red + blue, and white, showed no significant differences in the accumulation of total GSL contents. However, most of the individual GSL contents were lower in the red + blue light-exposed sprouts [36]. Similar results were obtained in this study; there were no significant changes in total GSL contents among the different LED-exposed kohlrabi-exposed sprouts. In addition, none of the individual GSLs showed the highest level under the red + blue LED light condition. These results show that most Brassica vegetables share the common individual GSL compounds. However different LED lights do not significantly affect the accumulation of individual and total GSL contents.

### 2.4. Accumulation of Carotenoid Content in Kohlrabi Sprouts Irradiated with Different LED Lights

**Figure 5:**
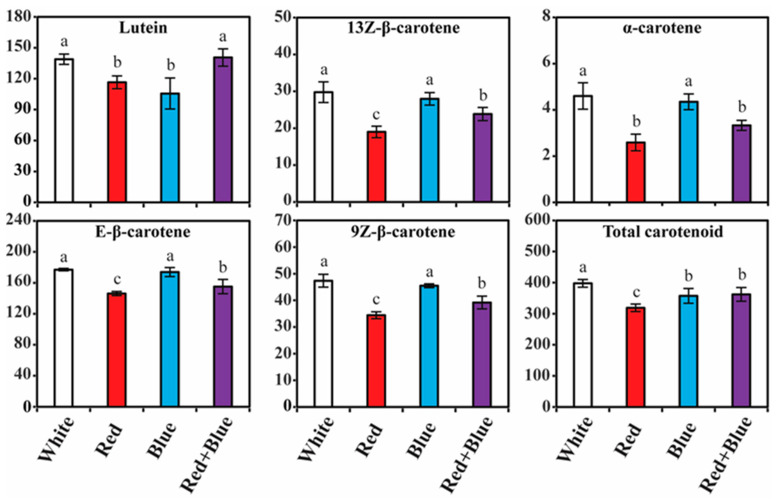
Carotenoid contents (µg/g DW) in kohlrabi sprouts irradiated with different LED lights. Samples were collected after 10 days of growth. Mean values marked with different alphabetical letters were significantly different (*p* < 0.05, ANOVA, DMRT).

Individual and total carotenoids varied significantly following the different LED light treatments (Figure 5 and Table S3). The total carotenoid contents in the different LED light conditions ranged from 318.71 to 397.70 μg/g DW. The highest amount of total carotenoid (μg/g DW) was accumulated in white LED light (397.70), followed by red + blue (362.12), blue (357.40), and red (318.71) LED light conditions. White LED light greatly influenced the total and individual carotenoid contents, except for the lutein content. The accumulation of Ε-β-carotene and lutein was significantly higher than that of other individual carotenoid compounds, irrespective of the different LED light treatments. The Ε-β-carotene contents in the different LED light conditions ranged from 146.23 to 177.11 μg/g DW (Figure 5 and Figure S1). The highest amount of Ε-β-carotene (177.11 μg/g DW) was achieved in white LED, which was 1.02-, 1.14-, and 1.21-times higher than the blue, red + blue, and red LED light treatments, respectively. The second most prevalent compound was lutein, which was highest in the red + blue LED light treatment as compared to the other LED treatments. The third most prevalent compound was 9Ζ-β-carotene, and it ranged from 34.42 to 47.35 μg/g DW, and the higher level was achieved in the white LED light condition, which was 1.04-, 1.21-, 1.37-times higher than the blue, red + blue, and red, respectively. Similarly, 13Z-β-carotene (29.76 μg/g DW) and α-carotene (4.60 μg/g DW) were highest in the white LED light treatment, whereas the lowest content was obtained in the red LED condition.

According to the results of our carotenoid compound analyses, irradiation with white LED light greatly influenced the total, as well as most of the individual carotenoid contents. This result was consistent with a previous study showing that exposure of red Chinese cabbage to white LED light enhanced the accumulation of total and individual carotenoids such as 13-cis-β-carotene, 9-cis-β-carotene, lutein, and β-carotene [39]. Similarly, carotenoid production increased when tartary buckwheat sprouts were exposed to white LED light [38]. In contrast, exposure of in vitro citrus juice sacs [72] and Chinese skullcap callus [42] to different LED lights showed a distinct increase in carotenoid biosynthesis. However, in the flavedo of citrus fruits, red LED light exposure increased carotenoid synthesis. The present and previous results show that the selection of suitable LED light for enhancing of total and individual carotenoids in plants might be species-specific. 

### 2.5. Metabolic Profiling

**Figure 6:**
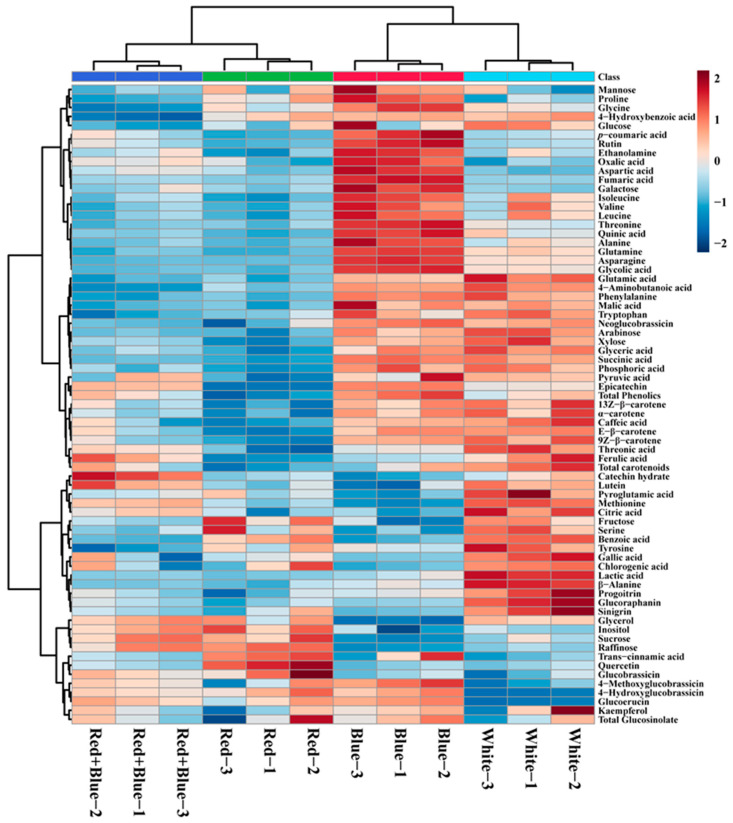
Heatmap showing the differences in the relative metabolite content in kohlrabi sprouts after exposure to different LED light conditions. The blue color represents an increase in the metabolite content, and the red color denotes a decrease in the metabolite content.

GC-TOF-MS and HPLC analyses of kohlrabi sprouts treated with different LED light sources identified 71 metabolites (amines, amino acids, carotenoids, GSLs, organic acids, phenolic acids, sugar alcohols, and sugars) (Figures S1 and S2). A heat map analysis of these identified metabolites showed that most of the metabolites were higher in blue and white than in those treated with red and red + blue LED lights (Figure 6). Tricarboxylic acid (TCA) cycle intermediates, such as glyceric acid, malic acid, and pyruvic acid, were highest in sprouts treated with blue and white LED lights. The highest levels of citric acid and threonic acid were observed in the white and red + blue LED lights, whereas the threonic acid content was highest in the white and red + blue LED lights. The levels of organic acids, such as lactic acid were highest under white LED light, whereas the levels of oxalic acid, aspartic acid, fumaric acid, and quinic acid were significantly higher only under the blue LED light treatment. Similarly, most amino acid levels were highest under white LED light, followed by blue LED light. The proline level was highest only under blue LED light, whereas the pyroglutamic acid and β-alanine levels were highest only under white LED light. The serine and tyrosine levels were highest under white and red LED lights, whereas the methionine level was highest under white and red + blue LED lights. This result was consistent with the previous study in which *S. baicalensis* was treated with different LED lights; the blue light exposure showed the highest number of metabolites (amino acids, organic acids, and TCA cycle intermediates) [73]. In addition, it was also found that white LED slightly enhanced the sugars and sugar alcohols, whereas blue and red LED lights showed lower accumulations of these metabolites [73]. Similarly, our study showed that the white and blue LED accumulated the highest contents of sugars and sugar alcohols compared to those treated with red and red + blue LED lights. These results indicated that different LED lights increased the contents of individual primary and secondary metabolites. Significantly higher (*p* ≤ 0.05) levels of individual metabolites in kohlrabi sprouts treated with various LED lights are shown in Figure S1. The results of this study may be helpful for researchers in selecting a suitable LED light to enhance the specific metabolite content in kohlrabi sprouts.

**Figure 7:**
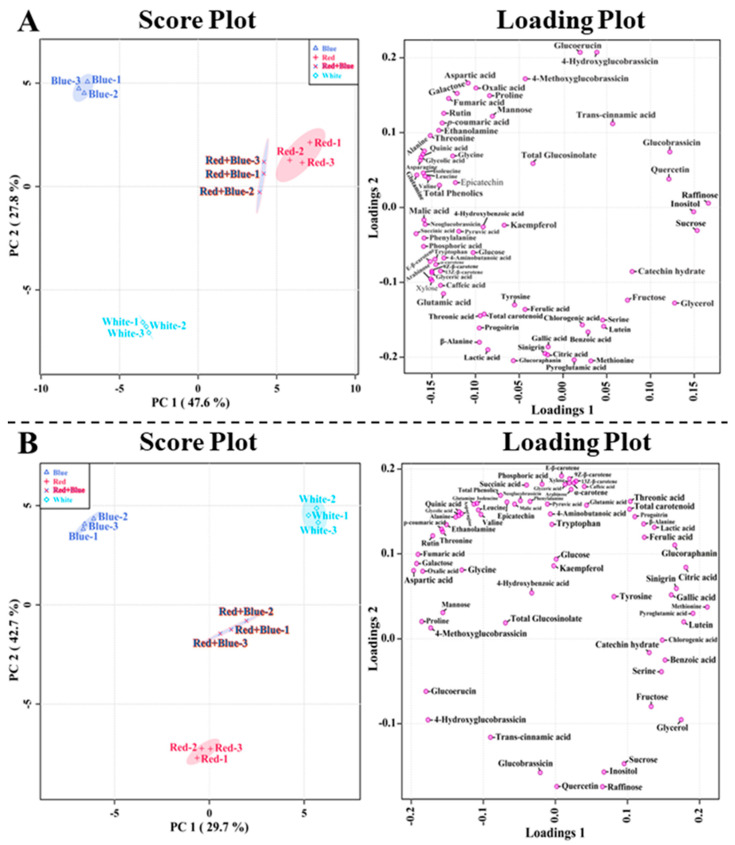
Score and loading plot of the PCA (**A**) and PLS-DA (**B**) models of the metabolites identified in kohlrabi sprouts after exposure to different LED light conditions.

The PCA results supported the metabolic profiling results obtained from kohlrabi sprouts treated with different LED lights (white, red, blue, and red + blue). The results of the PCA analysis showed that the metabolites obtained from the different LED light treatments showed 47.6% and 27.8% of the variance with a two-component analysis (principal components 1 (PC1) and PC2), respectively, as shown in Figure 7. In the PCA analysis, raffinose and sucrose showed positive eigenvector values of 0.16609 and 0.15329, respectively, whereas succinic acid, glutamine, asparagine, glycolic acid, and quinic acid showed negative eigenvector values of −0.16788, −0.16708, −0.1631, −0.16203, and −0.16018, respectively. The PCA results showed that all LED lights exhibited clear separation. A similar result was obtained in the PLS-DA analysis, which showed a clear separation in all LED treatments in its two-component analysis with 29.7% and 42.7% of the variance, respectively (Figure 7). This result supports the heatmap results, which showed that different LED lights increased the contents of individual metabolites, thus forming a separate group in the PCA and PLS-DA models. 

**Figure 8:**
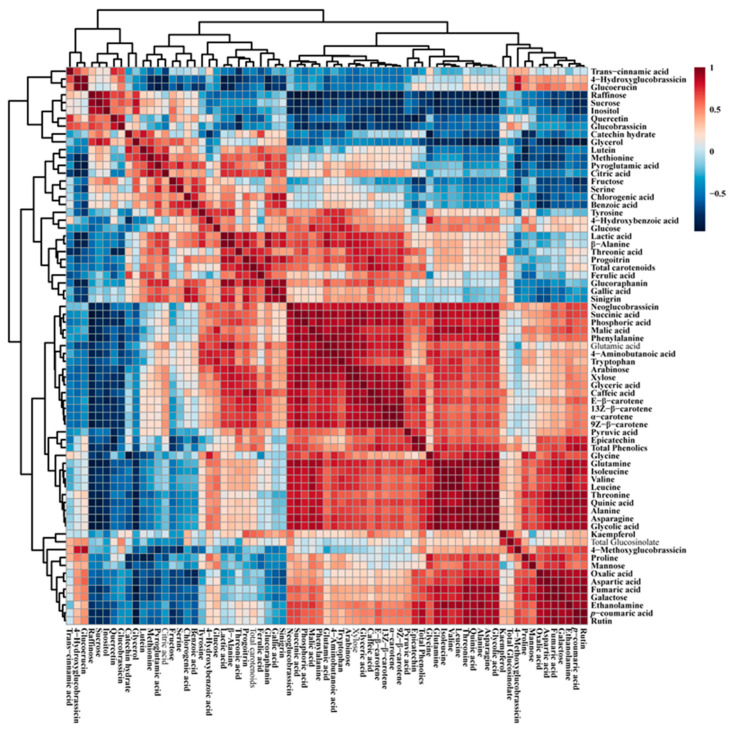
Correlation matrix of metabolites identified in kohlrabi sprouts after exposure to different LED light conditions. Each colored box represents a Pearson’s correlation coefficient for a pair of compounds, and the value of the correlation coefficient is indicated by the intensity of the blue or red color, as shown on the color scale.

Correlation analysis of the identified metabolites obtained from kohlrabi sprouts treated with different LED lights was performed using Pearson’s correlation (Figure 8). Phenylalanine, derived from the shikimate biosynthetic pathway, was positively correlated with the total carotenoid (*r* = 0.45423, *p* = 0.13796), total GSLs (*r* = 0.010209, *p* = 0.97488), and total phenolic contents (*r* = 057357, *p* = 0.051193). In addition, phenylalanine was also positively correlated with the highest levels of phenylpropanoid compounds in LED-treated kohlrabi sprouts, such as rutin (*r* = 0.57631, *p* = 0.049839) and epicatechin (*r* = 0.46348, *p* = 0.12912), which are intermediate compounds in the phenylpropanoid biosynthetic pathway. In addition, phenylalanine also showed a positive correlation with intermediate compounds in the GSL biosynthetic pathway, such as progoitrin, glucoraphanin, sinigrin, and neoglucobrassicin, whereas it was negatively correlated with 4-hydroxyglucobrassicin, 4-methoxyglucobrassicin, glucoerucin, and glucobrassicin. Furthermore, TCA cycle intermediates, such as malic acid (*r* = 0.64368, *p* = 0.60989) and succinic acid (*r* = 0.64428, *p* = 0.45572), were positively correlated with citric acid. Sucrose was positively correlated with most phenolic compounds, such as catechin hydrate, chlorogenic acid, trans-cinnamic acid, and quercetin, as well as sugar compounds, such as fructose, inositol, and raffinose.

**Figure 9:**
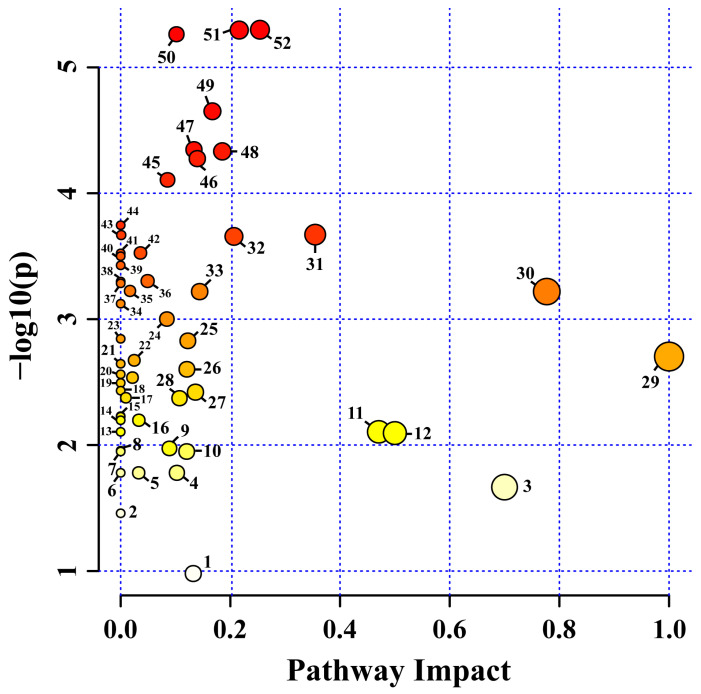
Identified metabolites and their pathway impact on kohlrabi sprouts after exposure to different LED light conditions. (1) Stilbenoid, diarylheptanoid and gingerol biosynthesis, (2) Terpenoid backbone biosynthesis, (3) Flavone and flavonol biosynthesis, (4) Inositol phosphate metabolism, (5) Phosphatidylinositol signaling system, (6) Ascorbate and aldarate metabolism, (7) Indole alkaloid biosynthesis, (8) Valine, leucine and isoleucine degradation, (9) Starch and sucrose metabolism, (10) Tryptophan metabolism, (11) Phenylalanine metabolism, (12) Isoquinoline alkaloid biosynthesis, (13) Tropane, piperidine and pyridine alkaloid biosynthesis, (14) Propanoate metabolism, (15) Pentose and glucuronate interconversions, (16) Sulfur metabolism, (17) Glycerophospholipid metabolism, (18) Purine metabolism, (19) Selenocompound metabolism, (20) Nitrogen metabolism, (21) Fatty acid degradation, (22) Flavonoid biosynthesis, (23) Nicotinate and nicotinamide metabolism, (24) Pantothenate and CoA biosynthesis, (25) Galactose metabolism, (26) Glycolysis/Gluconeogenesis, (27) Butanoate metabolism, (28) Valine, leucine and isoleucine biosynthesis, (29) Biosynthesis of secondary metabolites—unclassified, (30) Alanine, aspartate and glutamate metabolism, (31) Glycine, serine and threonine metabolism, (32) Glyoxylate and dicarboxylate metabolism, (33) Arginine and proline metabolism, (34) Monobactam biosynthesis, (35) Glycerolipid metabolism, (36) Carotenoid biosynthesis, (37) Pyrimidine metabolism, (38) Thiamine metabolism, (39) Cyanoamino acid metabolism, (40) Glucosinolate biosynthesis, (41) Aminoacyl-tRNA biosynthesis, (42) Carbon fixation in photosynthetic organisms, (43) Ubiquinone and other terpenoid-quinone biosynthesis, (44) Amino sugar and nucleotide sugar metabolism, (45) Arginine biosynthesis, (46) Cysteine and methionine metabolism, (47) Glutathione metabolism, (48) Citrate cycle (TCA cycle), (49) Pyruvate metabolism, (50) Phenylpropanoid biosynthesis, (51) Tyrosine metabolism, and (52) Beta-alanine metabolism.

Fifty-two pathways were identified in kohlrabi sprouts exposed to various LED lights. For the pathway analysis, the *Arabidopsis thaliana* Kyoto Encyclopedia of Genes and Genomes (KEGG) metabolic pathway was used as a source of pathway libraries (Figure 9). Among these, 36 pathways were affected in the present study (Table S4) when kohlrabi sprouts were exposed to different LED lights. Among these, fourteen pathways were involved in amino acid synthesis and metabolism, whereas six of these pathways were related to carbohydrate metabolism. In addition, other pathways, such as butanoate metabolism, glutathione metabolism, and isoquinoline alkaloid biosynthesis, were also affected. Similarly, plants grown under various irrigation regimes, such as chia seeds [74] and cannabis seeds grown under two different conditions [75], exhibited that the identified metabolites have the greatest impact on organic compounds, especially nutraceutical relevance. In detail, in arginine biosynthesis, the intermediate amino acids such as L-glutamate and β-alanine were significantly highest in the blue and white LED light treatments when compared to the other LED light treatments, respectively. In addition, in tyrosine metabolism, the content of L-tyrosine was highest in the white LED light treatment, whereas the L-glutamate, glycine, and 5-oxoproline contents were highest in the white, blue, and white LED treatments, respectively. Moreover, in the cysteine and methionine metabolites, the intermediate amino acids such as L-aspartate and L-methionine were highest in the blue and white LED light treatments, respectively. The arginine biosynthesis intermediates such as L-glutamine, L-glutamate, and L-aspartate were highest in the blue, white, and blue LED treatments, respectively. In contrast, in the glycine, serine, and threonine metabolisms, most of the amino acid intermediates such as L-aspartate, L-threonine, glycine, and L-tryptophan were highest in the blue LED treatment when compared to the other LED treatments. Similarly, in the thiamine metabolism and alanine, aspartate, and glutamate metabolisms, most of the intermediate amino acids were significantly highest in the blue LED treatment, whereas in the arginine and proline metabolisms, the amino acid intermediates such as the L-proline and L-glutamate contents were highest in the blue and white lights, respectively. The amino acid intermediates present in the lysine biosynthesis were highest in the blue light, whereas the phenylalanine, tyrosine, and tryptophan biosynthesis amino acids intermediates were highest in the blue and white lights. However, in the valine, leucine, and isoleucine biosynthesis, all the intermediate amino acids were highest in the blue LED treatment. In the phenylalanine metabolism, the L-phenylalanine content was highest in the blue light, whereas, in the tryptophan metabolism, the L-tryptophan content was highest in the white LED treatment. From this result, it is shown that the red and red + blue LED lights significantly affect amino acid synthesis and metabolism. Moving on to the butanoate metabolism, the intermediates such as pyruvate and succinate were highest in the blue LED treatment, whereas the intermediates such as L-glutamate and 4-aminobutanoate were highest in the white LED treatment. Similarly, in the glutathione metabolism, most of the intermediates were highest in the blue and white LED treatments, whereas the intermediates present in isoquinoline alkaloid biosynthesis were highest in the white LED treatment. From this result, it is shown that kohlrabi sprouts exposed to red and red + blue LED lights significantly affect the butanoate metabolism, glutathione metabolism, isoquinoline alkaloid biosynthesis, and amino acid synthesis and metabolism. These results indicate that the metabolites and their pathways were significantly affected by nutraceutical applications.

**Figure 10:**
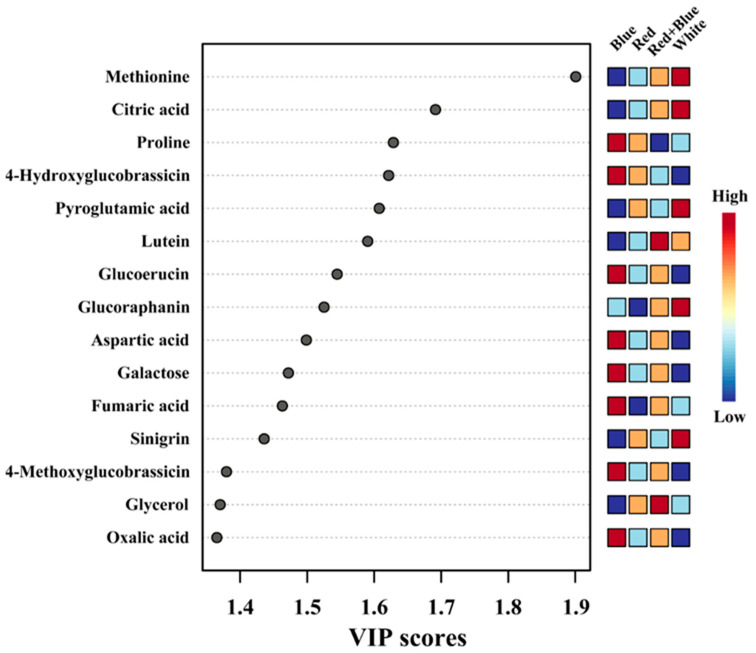
The main components, separating the kohlrabi sprouts after exposure to different LED light conditions, are based on the VIP scores attained through the PLS-DA model.

The VIP and Significance Analysis of Microarray (SAM) analyses were performed to identify the significant metabolites among the different LED light exposures. PLS-DA identified the most important metabolites based on the VIP score of the five-component model of >1. In total, 15 compounds were identified as important metabolites (VIP > 1) among kohlrabi sprouts exposed to different LEDs (Figure 10). In addition, SAM identified similar metabolites based on the d-value of the metabolites at a delta value of 2.6, a false discovery rate of 0.001, and a false positive of 5.37 (Figure S2).

## 3. Materials and Methods

### 3.1. Plant Materials

Red kohlrabi (Ruby Cap F_1_) seeds were purchased from Asia Co., Ltd. (Seoul, Republic of Korea). The experiment was carried out in the Department of Crop Science, Chungnam National University, Daejeon, Korea. One gram of seeds was sown in an 11 cm × 11 cm plastic pot containing vermiculite soil and kept in an LED growth chamber (Multi-room chamber HB302S-4, Hanbaek Scientific Co., Bucheon, Republic of Korea) at 25 °C under 8 h dark/16 h light photocycle and irradiated with white (449–551 nm), red (636 nm), blue (452 nm), and red + blue (457 and 636 nm). The LED spectrum was measured by using a spectrometer (Li-180, LI−COR, Lincoln, NE, USA) (Figure S3). LED lights had a flux rate of 90 µmol·m^−2^·s^−1^. The blue and red LED light treatments were monochromatic. The LED lights were purchased from PARUS LED Co., Ltd., Cheoan, Republic of Korea, which consisted of white (R/B = 6/12), red, and blue components. After 10 days, kohlrabi sprouts were harvested, and fresh weight, shoot length, and root length were measured. The harvested samples were freeze-dried at −80 °C for 72 h and ground into a fine powder for GC-TOF-MS and HPLC analyses. All the experiments were performed in triplicate.

### 3.2. Phenylpropanoid Extraction and HPLC Analysis

To the 100 mg powder sample, 2 mL of 80 % (*v*/*v*) methanol was added. The mixture was vortexed and sonicated for 1 h at 36 °C. Then, the samples were centrifuged at 12,000 rpm for 10 min at 4 °C, and the supernatant was collected. The supernatant was aliquoted into new tubes, filtered using 0.45 µm Whatman No. 42 filter paper, and stored in vials until HPLC analysis. The mobile phase, HPLC conditions, flow rate, and gradient program were similar to those described by Sathasivam et al. [76]. The phenylpropanoid contents were calculated according to the HPLC peak areas compared to the internal standard calibration curves. 

### 3.3. Glucosinolates Extraction and HPLC Analysis

For GSLs extraction, 100 mg of finely powdered samples were placed in a 2 mL tube, and mixed gently after adding 1.5 mL of 70 % (*v*/*v*) methanol. Subsequently, they were incubated in a water bath at 70 °C for 5 min for myrosinase inactivation and centrifuged at 12,000 rpm for 10 min. The supernatant was collected in a new 5 mL tube, and this process was repeated thrice to collect 4.5 mL of the supernatant. The collected supernatant was loaded into a mini-column filled with DEAE Sephadex A-25 and loaded with 2 mL of water. The end of the mini-column was blocked and desulfated by adding 75 μL of aryl sulfatase solution and left at room temperature for more than 16 h. After that, 0.5 mL of HPLC-grade water was used to elute the desulfo-GSLs. Finally, the solution was filtered through 0.45-μm filter paper. HPLC analysis was performed according to the method described by Sathasivam et al. [76]. 

### 3.4. Carotenoids Extraction and HPLC Analysis

The extraction and quantification of carotenoids were performed as described previously [77,78]. To extract carotenoids, 100 mg of kohlrabi sprout sample and 3 mL of ethanol containing 0.1 % ascorbic acid (*w*/*v*) were added and incubated for 5 min in a water bath at 85 °C. Next, 120 μL of 80 % potassium hydroxide (*w*/*v*) were added. After vortexing and incubating at 80 °C for 10 min, the sample was transferred to ice, and 0.1 mL of ethanol containing β-apo-8′-carotenal (25.0 μg mL^−1^) was added as an internal standard. After 1.5 mL of cold deionized water were added, the extraction was carried out twice by adding 1.5 mL of hexane, centrifuging at 1200× *g*, and collecting the supernatant. The extract was dried and resuspended in 50:50 (*v*/*v*) dichloromethane/methanol. HPLC analysis was performed according to the method described by Sathasivam et al. [79,80] (Figure S4). The carotenoid contents were calculated according to the HPLC peak areas compared to the internal standard calibration curves.

### 3.5. GC-TOF-MS Analysis

Hydrophilic compounds were extracted and analyzed according to the method described by Sathasivam et al. [81]. Briefly, 10 mg of finely powdered samples were placed in a 2 mL tube and mixed gently after adding 1 mL of methanol:chloroform:water (2.5:1:1, *v*/*v*/*v*) and 60 μL of ribitol (200 μg/mL in methanol) as an internal standard. The water/methanol phase was completely evaporated in a centrifugal concentrator (CC-105, TOMY, Tokyo, Japan) for 3 h and freeze-dried at -80 °C for at least 16 h. For derivatization, the samples were treated with 80 μL of methoxyamine hydrochloride in pyridine (20 mg/mL) and 80 μL of *N*-methyl-*N*-(trimethylsilyl)trifluoroacetamide. The derivatized samples were analyzed using an Agilent 7890A GC (Agilent Technologies, Santa Clara, CA, USA) connected with a Pegasus TOF-MS (Leco, St. Joseph, MI, USA). The Rtx-5MS column (30 m × 0.25 mm, 0.25-μm i.d. film thickness; Restek, Bellefonte, PA, USA) was used to separate the hydrophilic compounds. The GC-TOFMS analysis was performed as described in a previous study [66]. Peaks were identified based on mass spectral data compared to the standards, NIST 11, Wiley 9, and in-house libraries. For quantification, the relative ratio of the peak area of the compounds to the peak area of the internal standard was obtained based on the selected ions (Table S5). The representative GC-TOF-MS chromatogram is shown in Figure S5. We confirmed the hydrophilic compounds based on the standards (MSI level 1) in targeted metabolite profiling [82]. ChromaTOF software (LECO, St. Joseph, MO, USA, version 5.10) was used to identify the hydrophilic compounds in kohlrabi. The Chroma TOF software package was used to extract raw peaks, filter and calibrate data baselines, align peaks, perform deconvolution analysis, identify peaks, and integrate peak areas. The experiments were performed in triplicate, and the collected sprouts from the three independent replicates were used for GC-TOF-MS analysis for the identification of hydrophilic compounds.

### 3.6. Statistical Analysis

Phenylpropanoid, GSLs, and carotenoid data were analyzed using SPSS version 26 (SPSS, Chicago, IL, USA) following Duncan’s multiple range test (DMRT). All data were calculated in triplicate. PCA, PLS-DA, correlation analysis, hierarchical clustering, heat map, pathway impact, and variable importance in projection (VIP) of the 71 identified metabolites after exposure of kohlrabi sprouts to different LED conditions were performed using MetaboAnalyst 5.0 (http://www.metaboanalyst.ca/, accessed on 15 September 2022) with autoscaling.

## 4. Conclusions

The present study aimed to analyze the effects of different LED lights on the composition of primary and secondary metabolites in kohlrabi sprouts. The results revealed that blue LED light was more suitable for sprout growth and for increasing the phenylpropanoid and GSLs levels in kohlrabi sprouts. In contrast, exposure to white LED light was the most effective for enhancing carotenoid accumulation. The primary metabolite analysis showed that most of the metabolic precursors of secondary metabolites, such as amino acids, TCA intermediates, sugar, and sugar alcohol, were increased under blue and white lights. The overall study results suggest that the effect of different LED lights and wavelengths on plant primary and secondary metabolite accumulations may be dependent on the plant species, especially based on the cell and tissue types and organ. In addition, this study provides valuable information for the enhancement of specific primary and secondary metabolites in red kohlrabies using various LED lights. However, in the future, further research with differences in altering the light quality ratios is recommended to better understand the relationship between light quality and light intensity regarding primary and secondary metabolite concentrations. These research findings will also be further helpful to the researchers who continue to investigate the effects of various LED lights on plant morphology and the production of nutritionally valuable metabolites in the specialty crop production system. 

## Data Availability

Data reported are available in the Supplementary Materials.

## Supplementary Materials

The following supporting information can be downloaded at: https://www.mdpi.com/article/10.3390/plants12061296/s1: Figure S1: Individual metabolites that are significantly (*p* ≤ 0.05) higher in kohlrabi sprouts irradiated with different LED lights; Figure S2: The important metabolites identified through SAM; Figure S3: Spectrum analysis of different LED light sources; Figure S4: HPLC chromatogram of carotenoid compounds from kohlrabi treated with white LED. Peak: 1, lutein; 2, trans-β-Apo-8′-carotenal (internal standard); 3, 13Z-β-carotene; 4, α-carotene; 5, E-β-carotene; 6, 9Z-β-carotene; Figure S5: GC-TOF-MS analytical ion chromatogram (AIC) of hydrophilic compounds extracted from Kohlrabi treated with white LED. 1, Pyruvic acid; 2, Lactic acid; 3, Glycolic acid; 4, Alanine; 5, Oxalic acid; 6, Valine; 7, Serine 1; 8, Ethanolamine; 9, Phosphoric acid; 10, Glycerol; 11, Leucine; 12, Isoleucine; 13, Proline; 14, Glycine; 15, Succinic acid; 16, Glyceric acid; 17, Fumaric acid; 18, Serine 2; 19, Threonine; 20, β-Alanine; 21, Malic acid; 22, Aspartic acid; 23, Methionine; 24, Pyroglutamic acid; 25, 4-Aminobutanoic acid; 26, Threonic acid; 27, Glutamic acid; 28, Phenylalanine; 29, Xylose 1; 30, Xylose 2; 31, Arabinose; 32, Asparagine; 33, Ribitol; 34, Glutamine; 35, Citric acid; 36, Quinic acid; 37, Fructose 1; 38, Fructose 2; 39, Mannose; 40, Galactose; 41, Glucose 1; 42, Glucose 2; 43, Tyrosine; 44, Inositol; 45, Tryptophan; 46, Sucrose; 47, Raffinose; Table S1: Phenylpropanoid content (µg/g DW) in kohlrabi sprouts irradiated with different LED lights; Table S2: Glucosinolate content (µg/g DW) in kohlrabi sprouts irradiated with different LED lights; Table S3: Carotenoid content (µg/g dry weight) in kohlrabi sprouts irradiated with different LED lights; Table S4: Metabolic pathways where the identified metabolites are involved in kohlrabi sprouts irradiated with different LED lights; Table S5: Retention times (RTs), relative retention times (RRTs) and quantification ion data of hydrophilic compounds in Kohlrabi.
