# Supplementary material for: Metabolic Profiling of Primary and Secondary Metabolites in Kohlrabi (Brassica oleracea var. gongylodes) Sprouts Exposed to Different Light-Emitting Diodes
Source: Plants (Basel). 2023 Mar 13;12(6):1296. doi: 10.3390/plants12061296 (PMC10057582; doi:10.3390/plants12061296)
Supplement: Supplementary file 1 [file plants-12-01296-s001.zip › Figure S1, S2, and S4.pdf]

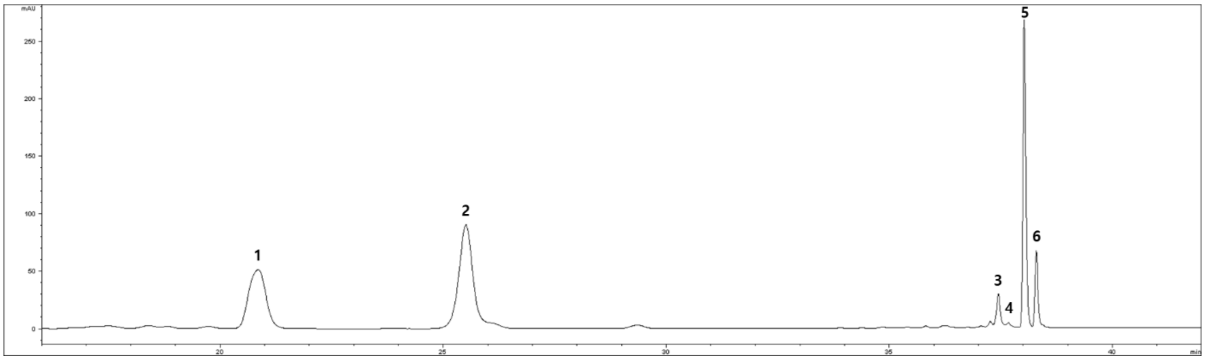

Figure S1: HPLC chromatogram of carotenoid compounds from Kohlrabi treated with white LED. Peak: 1, lutein; 2, trans-β-Apo-8′-carotenal (internal standard); 3, 13Z-β-carotene; 4, α-carotene; 5, E-β-carotene; 6, 9Z-β-carotene.

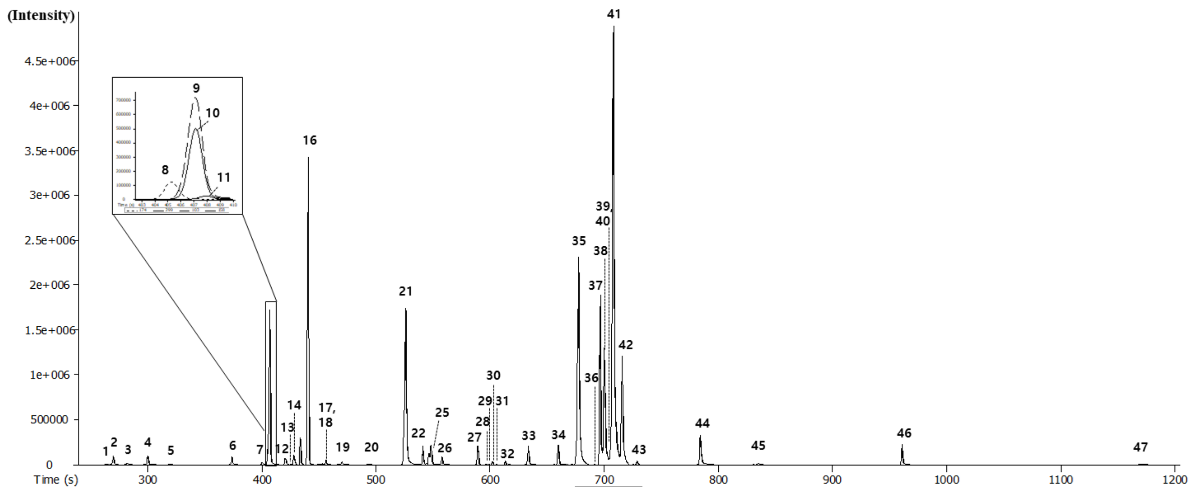

Figure S2: GC-TOF-MS analytical ion chromatogram (AIC) of hydrophilic compounds extracted from Kohlrabi treated with white LED. 1, Pyruvic acid; 2, Lactic acid; 3, Glycolic acid; 4, Alanine; 5, Oxalic acid; 6, Valine; 7, Serine 1; 8, Ethanolamine; 9, Phosphoric acid; 10, Glycerol; 11, Leucine; 12, Isoleucine; 13, Proline; 14, Glycine; 15, Succinic acid; 16, Glyceric acid; 17, Fumaric acid; 18, Serine 2; 19, Threonine; 20, β-Alanine; 21, Malic acid; 22, Aspartic acid; 23, Methionine; 24, Pyroglutamic acid; 25, 4-Aminobutanoic acid; 26, Threonic acid; 27, Glutamic acid; 28, Phenylalanine; 29, Xylose 1; 30, Xylose 2; 31, Arabinose; 32, Asparagine; 33, Ribitol; 34, Glutamine; 35, Citric acid; 36, Quinic acid; 37, Fructose 1; 38, Fructose 2; 39, Mannose; 40, Galactose; 41, Glucose 1; 42, Glucose 2; 43, Tyrosine; 44, Inositol; 45, Tryptophan; 46, Sucrose; 47, Raffinose.

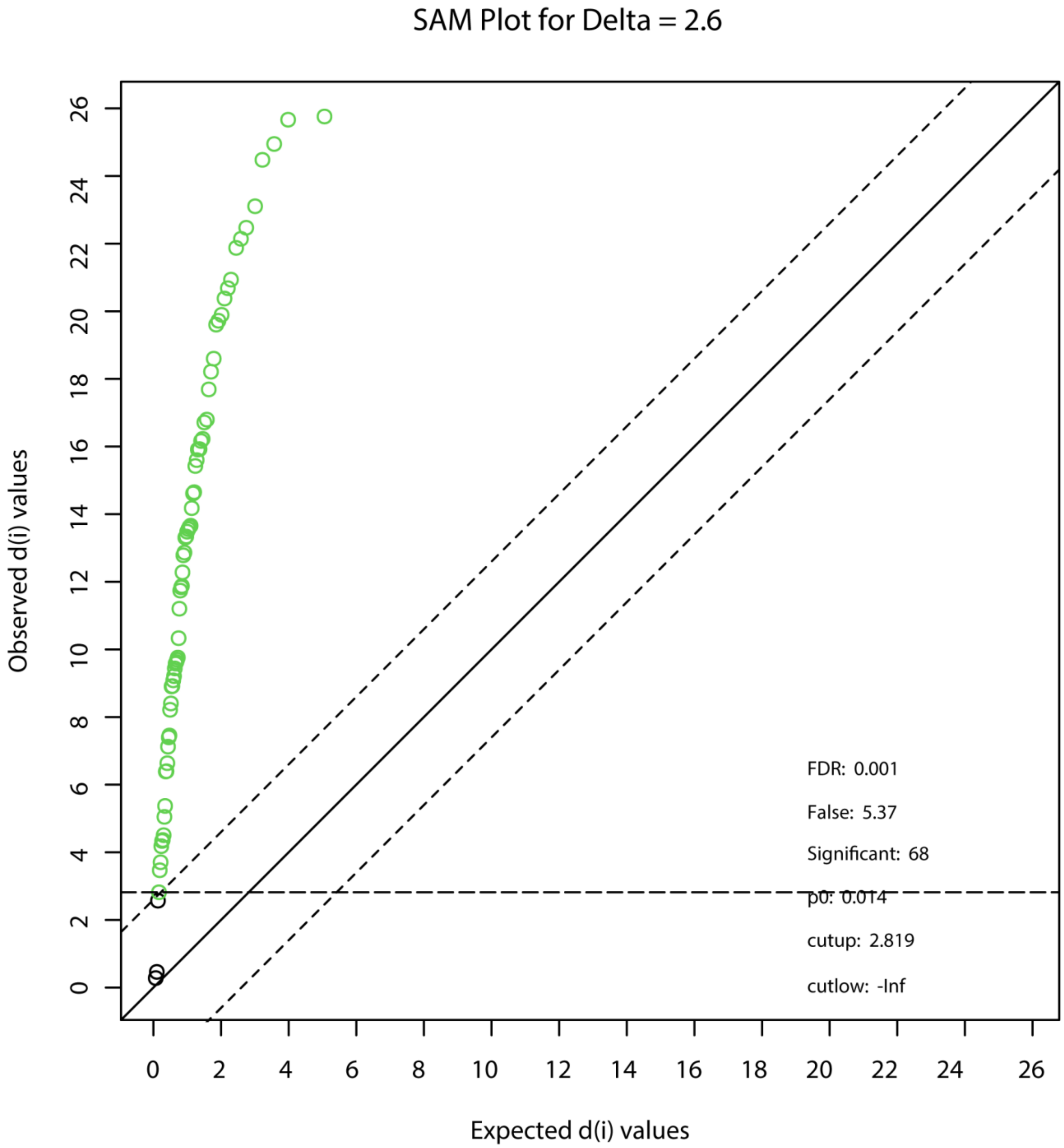

Figure S4: The important metabolites identified through SAM.
