# Supplementary material for: Metabolic Profiling of Primary and Secondary Metabolites in Kohlrabi (Brassica oleracea var. gongylodes) Sprouts Exposed to Different Light-Emitting Diodes
Source: Plants (Basel). 2023 Mar 13;12(6):1296. doi: 10.3390/plants12061296 (PMC10057582; doi:10.3390/plants12061296)
Supplement: Supplementary file 1 [file plants-12-01296-s001.zip › Table S1, S2, S3, and S5.pdf]

Table S1. Phenylpropanoid content (μg/g DW) in kohlrabi sprouts irradiated with different LED lights. Samples were collected after 10 days of growth.

Compounds	White	Red	Blue	Red+Blue
Gallic acid	31.60±2.51 a	21.93±1.49 b	17.88±0.34 b	19.40±8.21 b
4-hydroxybenzoic acid	38.50±2.08 a	35.60±3.13 a	38.21±0.73 a	18.59±1.38 b
Catechin hydrate	110.80±8.12 b	98.29±4.51 c	86.87±5.70 c	136.12±7.21 a
Chlorogenic acid	80.96±0.76 a	76.11±7.28 a	68.95±0.90 b	72.15±6.96 ab
Caffeic acid	66.84±3.35 a	47.00±2.20 b	61.65±0.83 a	53.05±6.29 b
Epicatechin	578.45±12.14 c	305.63±4.67 d	743.60±6.27 a	665.32±8.62 b
p-coumaric acid	109.84±2.93 b	97.14±1.73 c	151.71±8.49 a	113.41±7.62 b
Ferulic acid	32.40±3.62 a	18.84±0.88 b	24.15±0.58 b	31.23±3.63 a
Benzoic acid	174.13±3.44 a	155.50±12.99 b	97.28±8.57 c	98.87±3.04 c
Rutin	1002.17±6.20 b	974.60±6.27 c	1135.77±14.68 a	1015.35±17.70 b
Trans-cinnamic acid	13.58±1.28 b	22.01±0.86 a	17.97±5.62 ab	15.11±0.84 b
Quercetin	89.46±9.09 b	166.87±13.73 a	85.82±8.71 b	93.03±7.65 b
Kaempferol	119.59±17.87 a	110.17±10.40 a	119.62±3.05 a	115.10±5.54 a
Total	2448.3±74.39 b	2129.7±70.14 c	2649.5±64.47 a	2446.7±84.69 b

Mean values marked with different alphabetical letters were significantly different ($p < 0.05$, ANOVA, DMRT).

Table S2. Glucosinolate content (µg/g DW) in kohlrabi sprouts irradiated with different LED lights. Samples were collected after 10 days of growth.

**Compounds**	**White**	**Red**	**Blue**	**Red+Blue**
Progoitrin	3.41±0.25 a	2.43±0.32 b	2.78±0.05 b	2.65±0.14 b
Glucoraphanin	4.94±0.44 a	2.23±0.52 b	2.34±0.07 b	2.60±0.27 b
Sinigrin	4.85±0.40 a	3.75±0.55 b	3.36±0.06 b	3.57±0.16 b
4-Hydroxyglucobrassicin	0.49±0.09 b	3.77±0.86 a	3.90±0.48 a	3.15±0.20 a
Glucoerucin	2.47±0.07 b	3.87±0.46 a	4.34±0.13 a	4.07±0.20 a
Glucobrassicin	3.93±0.25 b	4.66±0.38 a	4.06±0.11 b	4.35±0.11 ab
4-Methoxyglucobrassicin	4.00±0.19 b	4.35±0.47 b	4.89±0.15 a	4.53±0.08 ab
Neoglucobrassicin	2.92±0.06 a	2.44±0.26 b	3.02±0.05 a	2.46±0.02 b
Total	27.01±1.75 a	27.50±3.82 a	28.69±1.1 a	27.38±1.18 a

Mean values marked with different alphabetical letters were significantly different ($p < 0.05$, ANOVA, DMRT).

Table S3. Carotenoid content (μg/g dry weight) in kohlrabi sprouts irradiated with different LED lights. Samples were collected after 10 days of growth.

**Compounds**	**White**	**Red**	**Blue**	**Red+Blue**
Lutein	138.88±4.99 a	116.48±6.24 b	105.57±15.05 b	140.56±8.44 a
13Z-β-carotene	29.76±2.79 a	18.99±1.56 c	27.96±1.69 a	23.84±1.78 b
α-carotene	4.60±0.57 a	2.59±0.36 b	4.35±0.34 a	3.33±0.22 b
E-β-carotene	177.11±1.55 a	146.23±2.67 c	174.04±5.87 a	155.21±9.21 b
9Z-β-carotene	47.35±2.41 a	34.42±1.29 c	45.48±0.73 a	39.18±2.38 b
Total	397.70±12.31 a	318.71±12.12 c	357.40±23.68 b	362.12±22.03 b

Mean values marked with different alphabetical letters were significantly different ($p < 0.05$, ANOVA, DMRT).

Table S5. Retention times (RTs), relative retention times (RRTs) and quantification ion data of hydrophilic compounds in Kohlrabi.

No.	Compounds	RT (s) [a]	RRT [b]	Quant ion [c]
1	Pyruvic acid	263.9	0.415656	174
2	Lactic acid	269.6	0.424634	147
3	Glycolic acid	282.0	0.444164	147
4	Alanine	300.2	0.47283	116
5	Oxalic acid	319.5	0.503229	147
6	Valine	373.9	0.588912	144
7	Serine 1	400.3	0.630493	116
8	Ethanolamine	405.4	0.638526	174
9	Phosphoric acid	406.9	0.640888	299
10	Glycerol	407.4	0.641676	103
11	Leucine	407.8	0.642306	158
12	Isoleucine	420.8	0.662782	158
13	Proline	425.5	0.670184	142
14	Glycine	429.1	0.675854	174
15	Succinic acid	433.8	0.683257	147
16	Glyceric acid	440.1	0.69318	147
17	Fumaric acid	454.5	0.715861	245
18	Serine 2	457.5	0.720586	116
19	Threonine	471.5	0.742637	219
20	β-Alanine	496.9	0.782643	248
21	Malic acid	526.0	0.828477	147
22	Aspartic acid	542.6	0.854623	232
23	Methionine	545.2	0.858718	176
24	Pyroglutamic acid	548.4	0.863758	156
25	4-Aminobutanoic acid	549.7	0.865806	174
26	Threonic acid	559.1	0.880611	147
27	Glutamic acid	591.0	0.930855	246
28	Phenylalanine	598.0	0.941881	218
29	Xylose 1	599.9	0.944873	103
30	Xylose 2	603.5	0.950543	103
31	Arabinose	606.9	0.955899	103
32	Asparagine	615.1	0.968814	116
33	Ribitol	634.9	1	217
34	Glutamine	661.3	1.041581	156
35	Citric acid	678.0	1.067885	273

36	Quinic acid	692.9	1.091353	345
37	Fructose 1	698.6	1.100331	103
38	Fructose 2	702.5	1.106473	103
39	Mannose	703.0	1.107261	319
40	Galactose	705.1	1.110569	319
41	Glucose 1	709.9	1.118129	319
42	Glucose 2	717.5	1.130099	319
43	Tyrosine	730.5	1.150575	218
44	Inositol	786.3	1.238463	305
45	Tryptophan	836.2	1.317058	202
46	Sucrose	963.1	1.516932	217
47	Raffinose	1177.1	1.853993	361

[a] Retention time (sec)

[b] Relative retention time (retention time of the analyte/retention time of the ribitol)

[c] Quantification mass ion
